# A retrospective cross-sectional study on newborn screening and prevalence of disorders among UAE population

**DOI:** 10.3389/fped.2026.1788876

**Published:** 2026-06-10

**Authors:** Khadija Shafique, Afsheen Raza, Ariba Naushad, Fathima Hussain, Duaa Walid, Laila Wareth, Ayman W. El-Hattab, Rania Nabil Bedair, Rania Al Dweik, Najwane Said Sadier

**Affiliations:** 1 Department of Genetics and Genomics, UAE University (UAEU), Al Ain, United Arab Emirates; 2 Department of Biomedical Sciences, College of Health Sciences, Abu Dhabi University, Abu Dhabi, United Arab Emirates; 3 Cancer Research Institute, Office of Research and Sponsored Programs, Abu Dhabi University, Abu Dhabi, United Arab Emirates; 4 Genetics and Rare Disease Center, Burjeel Medical City, Abu Dhabi, United Arab Emirates; 5 National Reference Laboratory, M42, Abu Dhabi, United Arab Emirates; 6 Department of Medical Sciences, College of Medical and Health Sciences, Khalifa University, Abu Dhabi, United Arab Emirates; 7 Medical Research Institute, Alexandria University, Alexandria, Egypt; 8 Department of Public Health, College of Health Sciences, Abu Dhabi University, Abu Dhabi, United Arab Emirates; 9 Sky Health Pharma, Abu Dhabi, United Arab Emirates; 10 Neuroscience Research Center, Faculty of Medical Sciences, Lebanese University, Beirut, Lebanon; 11 Deparment of Public Health, Dubai Health Authority, Dubai, United Arab Emirates

**Keywords:** congenital adrenal hyperplasia, congenital hypothyroidism, cystic fibrosis, G6PD deficiency, genetic and metabolic disorders, hemoglobinopathies, newborn screening

## Abstract

**Background:**

Newborn screening (NBS) is a crucial public health initiative designed to detect genetic, metabolic, and endocrine disorders in infants before clinical symptoms appear. Early detection enables timely intervention, reducing morbidity and mortality associated with congenital diseases.

**Objective:**

This study aimed to determine the prevalence of common disorders detected through newborn screening among the United Arab Emirates (UAE) population from year 2021 to 2023.

**Methods:**

A retrospective cross-sectional study was conducted using newborn screening data collected from the National Reference Laboratory (NRL), representing samples from 20 hospitals and laboratories across Abu Dhabi, Dubai, Sharjah, and Fujairah. Screening tests included hemoglobinopathies, aminoacidopathies, acylcarnitine disorders, cystic fibrosis, glucose-6-phosphate dehydrogenase (G6PD) deficiency, congenital hypothyroidism (CH), congenital adrenal hyperplasia (CAH), biotinidase deficiency, and galactose-1-phosphate uridylyltransferase (GALT) deficiency. Prevalence was calculated per 100,000 screened newborns using SPSS, excluding inconclusive and false-positive cases.

**Results:**

Of 29,290 newborns screened, 2,191 (7.4%) were positive for one or more disorders, and 698 (2.3%) were identified as hemoglobinopathy carriers. The highest prevalence was observed for G6PD deficiency (5,278 per 100,000 screened newborns), followed by cystic fibrosis (566 per 100,000 screened newborns), acylcarnitine disorders (518 per 100,000 screened newborns), and aminoacidopathies (505 per 100,000 screened newborns). Endocrine disorders, including CAH (160 per 100,000 screened newborns) and CH (116 per 100,000 screened newborns), were also detected. Hemoglobinopathies, particularly alpha-thalassemia and sickle cell anemia, represented the most common inherited blood disorders.

**Conclusion:**

The findings highlight a significant burden of G6PD deficiency and other metabolic disorders among newborns in the UAE. The study underscores the importance of continuous monitoring, expanded screening panels, and genetic counseling for carrier detection to improve neonatal outcomes and guide public health strategies.

## Introduction

Newborn screening is an initiative that was launched to screen, test and manage genetic disorders in infants (1). Each year, around 4 million infants are screened for genetic disorders around the globe as congenital disorders can often go unnoticed due to the lack of symptoms but can manifest later on during life. For example, infants with inherited adrenal hyperplasia start to show symptoms 1–4 weeks after birth (2). In lieu of this, around 3,400 children are early identified for the congenital diseases early on as a result of the screening tests, indicating the importance of newborn screening. The screening in most countries involves a heel-prick test for collection of blood within 24–48 h after birth to initiate testing for diagnosis of specific disorders and initiate intervention accordingly., but other tests such as pulse oximetry and hearing screen can also be used (1). The main aim of newborn screening programs is to make sure that infants are screened early for pre-symptomatic diagnosis, detection, and management of disorders in newborns, which could lead to severe complications or conditions if not diagnosed in earlier stages (3, 4). Countries around the world have made it mandatory for infants that have just been delivered to be screened for genetic disorders due to the high number of infants being born with these disorders each year. Countries that actively support newborn screening include U.S, Canada, Russia, Pakistan, Saudi Arabia, United Arab Emirates etc. In Africa (5),. In Africa, newborn screening for hemoglobinopathies has been expanded through programs such as the Consortium on Newborn Screening in Africa (CONSA), which has supported screening in several countries. These initiatives have aimed to detect hemoglobin disorders early, especially sickle cell disease. Sickle cell disease has remained the most important condition identified in these programs, with an estimated prevalence of about 1 in 365 Black/African American births and 1 in 16,000 births overall. *β*-thalassemia major has occurred in approximately 1 in 100,000 births globally, and HbH disease has been reported in about 1 in 20,000 births in high-prevalence regions (6). Newborn screening in UAE started back in January of 1995 where the first disorder to be screened was PKU. Later, the screening system expanded, and infants were screened for more disorders which included sickle cell disease, inherited adrenal hyperplasia and inherited hypothyroidism (7).

The need of newborn screening around the globe led to development of panels consisting of a list of laboratory tests required for newborn screening for genetic diseases at birth. Additionally, the panels are specific to each country and depend on the prevalence of certain disorders in that country (1). For example, test panel used in United States comprises of 61 disorders in total, including metabolic disorders such as Phenylketonuria (PKU), tyrosinemia, and citrullinemia, along with endocrinal disorders of either thyroid or adrenal glands, and hemoglobinopathies (thalassemia and sickle cell disease) disorders. Other rare, but serious conditions like X -linked adrenoleukodystrophy, Pompe disease, cystic fibrosis, galactosemia, and severe combined immunodeficiency (SCID) are also detected by using the newborn screening panel (8). Using this panel, 12,000 infants with a core disorder (such as sickle cell disease) are identified annually (9). In contrast to USA, UAE uses a different panel that screens for five different classes of disorders, including amino acid disorders, organic acid disorders, fatty acid disorders, endocrine disorders and hemoglobinopathies. Supplementary tests can also be done such as biotinidase deficiency and cystic fibrosis, depending on the requirement.

Newborn screening is a significant step taken by governments all over the world to tackle the rising prevalence of genetic diseases (10). According to the Dubai Health Authority (DHA), around 153,000 newborns are annually screened for inherited disorders in the UAE (9). It is postulated that these high number of cases may be due to the high rates of consanguinity in Arab populations, residing in UAE (11). Making newborn screening essential for early diagnosis and critical follow-up with confirmatory tests and intervention for newborns identified with congenital disorders during the screening process (12). Newborn screening test in UAE is compulsory including not only the heel prick test but also congenital heart disease screening, hearing screening and examination by doctor (13). Screening newborns is also important on a large-scale for public health as it allows pre-symptomatic diagnosis, detection, and management of disorders in newborns, which could lead to severe complications or conditions if not diagnosed in earlier stages (3). Table 1 summarizes the most commonly identified disorders through newborn screening programs worldwide, along with their inheritance patterns and prevalence across different populations. Recently, Abu Dhabi introduced the Enaya screening programs which includes not only the heel prick test but also congenital heart disease screening, hearing screening and examination by doctor (13). The aim of this study is to identify the prevalence of common disorders and variations detected via newborn screening in UAE population.

**Table 1:** Distribution of inherited disorders reported in newborn screening studies across various populations, highlighting disease type, inheritance pattern, and frequency.

Category	Example disorders	Inheritance pattern/description	Key findings/prevalence	References
Fatty acid oxidation disorders	MCAD deficiency, VLCAD deficiency, SCAD deficiency, Carnitine uptake defect, CPT2 deficiency, Glutaric acidemia type 2	Autosomal recessive. These disorders impair the body’s ability to break down fatty acids for energy, especially during fasting or stress.	Duarte galactosemia highest among Whites (8.9%), Hispanics (5.6%), Middle Eastern (4.6%); MCAD deficiency highest among Native Americans (13.9%) and Whites (9.4%); SCAD deficiency, MSUD, and 3-methylcrotonyl-CoA carboxylase deficiency highest among Middle Eastern	Feuchtbaum et al. (14)
Amino acid disorders	Homocystinuria, PKU, MSUD, Methionine S-adenosyl transferase deficiency, Argininemia	Autosomal recessive. Caused by enzyme deficiencies affecting amino acid metabolism, leading to toxic buildup and developmental issues if untreated.	Zhejiang, China: 1,861,262 newborns screened; most common—Hyperphenylalaninemia (1:11,349); 7 deaths, 2 developmental delays	Huang et al. (15)
Organic acid disorders	Isovaleric acidemia (IVA), Propionic acidemia (PA), Glutaric acidemia type 1 (GA1)	Autosomal recessive. Involve accumulation of organic acids in body fluids, leading to metabolic acidosis, vomiting, lethargy, and developmental delays.	Bahrain: 1:6,000 newborns with amino acid disorders; 21/25 affected infants had consanguineous parents	Golbahar et al., (16)
Endocrine disorders	Congenital Hypothyroidism (CH), Congenital Adrenal Hyperplasia (CAH)	CH: usually sporadic; CAH: autosomal recessive. Affect hormone synthesis, causing growth, sexual development, and metabolic abnormalities.	Saudi Arabia: 199,143 newborns tested; CH (1:3,375), CAH (1:7,965); high consanguinity rates	Mujamammi (4)
Inherited metabolic disorders (UAE study)	Aminoacidopathies, Organic acidemias, Biotinidase deficiency, FAO disorders	Mostly autosomal recessive. Group of enzyme deficiencies affecting energy and nutrient metabolism.	UAE (2011–2014): incidence 1:1,787 (citizens); 25% had affected relatives	Al-Jasmi et al., (17)
Haemoglobinopathies	Alpha-thalassemia, Beta-thalassemia, Sickle cell anemia	Autosomal recessive. Affect hemoglobin synthesis or structure, leading to anemia and related complications.	Abu Dhabi (2005): 22,000 infants screened; 342 positive cases; Emiratis 0.07%, Non-Emiratis 0.02%	Al Hosani et al., (18)
Cystic fibrosis	Cystic Fibrosis (CFTR gene mutation, *Δ*F508 most common)	Autosomal recessive. Affects chloride channels, leading to thick mucus in lungs and digestive tract, recurrent infections.	Birth prevalence: Caucasians (38.8/100,000), Native Americans (37.2/100,000); >2,000 CFTR variants	Feuchtbaum et al., (14)

## Material and methods

### Study sites and screening tests

A single-center cross-sectional retrospective study was conducted using the newborn screening data collected through National Reference Laboratory, Abu Dhabi, UAE from 2021 to 2023. The screening data represented 20 hospitals and laboratories across three different Emirates including Abu Dhabi, Dubai and Sharjah. The screening data included the following disorders-haemoglobinopathies, aminoacidopathies, acylcarnitine disorders, cystic fibrosis, glucose-6-phosphate-dehydrogenase, congenital hypothyroidism, congenital adrenal hyperplasia, biotinidase deficiency, galactose-1-phosphate uridylyltransferas. Newborn screening was performed using standardized analytical platforms depending on the disorder category. Tandem mass spectrometry (MS/MS) was used for the detection of amino acid disorders and acylcarnitine profiles. Hemoglobinopathies were screened using high-performance liquid chromatography (HPLC). Cystic fibrosis screening was based on immunoreactive trypsinogen (IRT) immunoassay. Endocrine disorders were screened using immunoassay-based methods. All analyses were conducted at the National Reference Laboratory (NRL). Table 2 summarizes the specific methods, key analytes measured, normal reference ranges, screening cut-off thresholds, confirmatory testing protocols, and initial management recommendations for all disorders included in the program. Cut-off values were established through laboratory-specific validation studies conducted by the National Reference Laboratory and adjusted for factors such as prematurity and recent blood transfusions to achieve optimal analytical sensitivity (>99%) and specificity.

**Table 2:** Newborn screening analytical methods, analytes, and Cut-off thresholds by disorder category (national reference laboratory).

Disorder category	Analytical platform	Key analytes	Normal range	Cut-off threshold (flag)	Confirmatory test
Hemoglobinopathies	HPLC (Variant NBS), Capillary Electrophoresis (CE), IEF	HbF, HbA, HbS, HbC, Hb Bart’s, HbA2	HbF: 70–90%, HbA: 10–30%, HbA2: <3%	Hb Bart’s ≥1–2% (*α*-thal trait); FS/FSC/FSA patterns; HbA <10%	DNA analysis, repeat HPLC
Amino acid disorders (PKU, MSUD, etc.)	Tandem Mass Spectrometry (MS/MS)	Phe, Leu, Met, Tyr, Cit	Phe: 31–83 μmol/L; Leu: 41–179 μmol/L	Phe ≥120 μmol/L (PKU); Leu ≥365 μmol/L (MSUD)	Plasma AA analysis, enzyme assay
Organic acidemias (MCAD, MMA, etc.)	MS/MS (acylcarnitines)	C8, C3DC + C4OH, C3acyl, Methylmalonyl	C8: 0.20–0.44 μmol/L	C8 ≥ 0.44 μmol/L (MCAD); C3DC ≥0.5 μmol/L (GA1)	Urine organic acids, acylcarnitine profile
Fatty acid oxidation disorders	MS/MS	C14:1, C16:1OH, C5OH	C14:1: 0.02–0.26 μmol/L	C14:1 ≥ 0.26 μmol/L (VLCAD); C16:1OH ≥0.11 μmol/L	Acylcarnitine profile, genetic testing
Endocrine (CH, CAH)	Immunoassay (DELFIA/ELISA), HPLC	TSH, T4, 17-OHP	TSH: 0–10 mU/L; 17-OHP: <20 ng/mL	TSH ≥10 mU/L (CH); 17-OHP ≥25 ng/mL (CAH)	Repeat TSH/T4; 17-OHP LC-MS/MS
Biotinidase deficiency	Enzymatic/colorimetric assay	Biotinidase activity	>10% activity	<10% residual activity	Repeat enzyme assay
Galactosemia	Enzymatic assay, MS/MS (Gal-1-P)	GALT enzyme, Galactose-1-P	GALT: >20% activity	GALT <5% activity; Gal-1-P elevated	GALT enzyme assay, genetic testing
Cystic fibrosis	Immunoassay (IRT), DNA	IRT	IRT <60 ng/mL	IRT ≥60 ng/mL + sweat Cl⁻ or CFTR mutations	Sweat chloride test (>60 mmol/L)

### Statistical analysis

Data was collected in Excel and prevalence of different disorders was calculated using cross tabs created in SPSS. The prevalence was calculated using total screened as denominator for each disorder and positives between 2021 and 2023 as nominator multiplied by 100,000 (overall prevalence per 100,000 births screened newborns). Screen-positive newborns for hemoglobinopathies were categorized into two groups: (1) affected cases, and (2) carriers, defined as infants with heterozygous or trait status detected through screening. Carrier status was reported separately from affected cases and was not included in the calculation of disease prevalence. Presumptive positives or false positives with no confirmatory follow-up results, invalid, borderline and inconclusive results were removed from analysis which were about 189 cases in total.

## Results

The prevalence of disorders detected during NBS screening among UAE population tested at National Reference Laboratory (NRL) between 2021 and 2023 is shown in Table 3. A total of 29,290 newborns were screened at NRL for Galactose-1-phosphate uridylyltransferase deficiency (GALT), Biotinidase deficiency, Congenital Adrenal Hyperplasia (CAH), Congenital hypothyroidism (CH), Cystic Fibrosis (CF), Aminoacidopathies, Hemoglobinopathies, Acylcarnitine disorders, and Glucose-6-phosphate dehydrogenase deficiency (G6PD). Out of the total number of screened newborns, 2,191 (7.5%) were screened positive and 698 (2.4%) were detected as carriers of hemoglobin disorder for specific variants. The highest prevalence was observed for glucose-6-phosphate dehydrogenase (G6PD) deficiency, with 1,546 (5.3%) positive cases (5,278 per 100,000 screened newborns) followed by Cystic fibrosis, Acylcarnitine and aminoacidopathies, with prevalences of positivity rates of 566 (0.57%), 518 (0.52%) and 505 (0.51%) per 100,000 screened newborns, respectively. Other detected conditions included congenital adrenal hyperplasia (positivity rate 160 per 100,000 screened newborns) (0.16%), hemoglobinopathies (positivity rate 218 per 100,000 screened newborns) (0.22%), congenital hypothyroidism (positivity rate 116 per 100,000 screened newborns) (0.12%), biotinidase deficiency (positivity rate 68 per 100,000 screened newborns) (0.07%), and galactose-1-phosphate uridylyl transferase deficiency (positivity rate 47 per 100,000 screened newborns) (0.05%). These findings demonstrate a significant burden of G6PD deficiency in the screened population, while most other conditions were relatively less frequent.

**Table 3:** Overall prevalence rate of each class of NBS disorder and count of total screened, positive, negative, and inconclusive cases among UAE population, 2021–2023.

Class of disorders	Total screened	Screened negative	Screened positive	Prevalence per 100,000
Aminoacidopathies	29,226	29,211	148	505
Biotinidase deficiency	29,290	29,271	20	68
Congenital Adrenal Hyperplasia	29,280	29,249	47	160
Congenital hypothyroidism	29,212	29,185	34	116
Cystic Fibrosis	29,232	29,084	166	566
Galactose-1-phosphate uridylyl transferase deficiency	29,289	29,276	14	47
Glucose-6-phosphate dehydrogenase deficiency	29,101	27,961	1,546	5,278
Hemoglobinopathies	29,198	28,876	64	218
Acylcarnitine disorders	29,197	29,103	152	518

Aminoacidopathies detected were categorized as argininosuccinic aciduria (ASA), maple syrup urine disease (MSUD), phenylketonuria (Classical PKU), tyrosinemia II; tyrosinemia III; transient hypertyrosinemia (negative succinylacetone) (TYR II, TYR III, and TH), tyrosinemia type 1 (positive succinylacetone) (TYR1), homocystinuria (cystathionine ß-synthase deficiency; CBSD), proximal urea cycle disease, and other amino acid disorders. A total of 148 cases were identified with the most frequent being other amino acid disorders (*n* = 66; 225 per 100,000 screened newborns), followed by CBSD (*n* = 9; 30 per 100,000 screened newborns), PKU (*n* = 7; 23 per 100,000 screened newborns), and argininemia (*n* = 4; 13 per 100,000 screened newborns). Less common conditions included ASA (*n* = 2; 6 per 100,000 screened newborns), MSUD (*n* = 2; 6 per 100,000 screened newborns), proximal urea cycle disease (*n* = 1; 3 per 100,000 screened newborns), tyrosinemia types II/III/transient hypertyrosinemia (*n* = 3; 10 per 100,000 screened newborns), and tyrosinemia type I (*n* = 2; 6 per 100,000 screened newborns) as stated in Table 4. These findings indicate that aminoacidopathies, though relatively less frequent compared to G6PD deficiency and cystic fibrosis, still represent an important target group for newborn screening programs in the UAE.

**Table 4:** Overall prevalence, prevalence per 100,000, and cases screened positive during NBS screening for each disorder among UAE population, 2021–2023.

Classes of disorders	Screened positive	Prevalence per 100,000
Aminoacidopathies		
Argininosuccinic aciduria (ASA)	2	6
Argininemia	4	13
Homocystinuria (CBSD)	9	30
Maple syrup urine disease (MSUD)	2	6
Other amino acid disorders	52	177
Phenylketonuria (Classical PKU)	7	23
Proximal urea cycle disease (PUCD)	1	3
Tyrosinemia II/III/transient hypertyrosinemia	3	10
Tyrosinemia type 1 (TYR1)	2	6
Other amino acid disorders	66	225
Endocrine disorders		
Congenital adrenal hyperplasia (CAH)	47	160
Congenital hypothyroidism (CH)	34	116
Hemoglobinopathies		
Alpha thalassemia	22	75
Beta thalassemia	8	27
HbC disease	2	6
HbD/beta thalassemia	5	17
HbH disease	2	6
Sickle cell anemia	16	54
Other Hb disorder	9	30
Acylcarnitine disorders		
CPT2/CACT deficiency	17	58
Carnitine palmitoyl transferase I deficiency (CPT-1)	1	3
Carnitine uptake deficiency (C0-carnitine)	4	13
Isovaleric acidemia (IVA)	15	51
Propionic and methylmalonic acidemia (PAMMA)	37	126
Short-chain acyl-CoA dehydrogenase (SCAD)	6	20
Glutaric aciduria type 1 (GA1)	2	6
Malonic aciduria (C3dc)	1	3
Other acylcarnitine disorders	69	235

For endocrine disorders, a total of 47 cases of congenital adrenal hyperplasia (CAH; 160 per 100,000 screened newborns) and 34 cases of congenital hypothyroidism (CH; 116 per 100,000 screened newborns) were detected. CAH was more frequent than CH in this cohort, highlighting the relevance of endocrine screening as part of the national newborn screening program. Different types of acylcarnitine disorders were also including Carnitine palmitoyl transferase I deficiency (CPT-1), carnitine palmitoyl transferase 2 deficiency or carnitine- acylcarnitine translocase deficiency (CPT2/CACT), carnitine uptake deficiency (C0-carnitine), short-chain acyl-CoA dehydrogenase (SCAD), isovaleric acidemia (IVA)C5-carnitine, propionic and methylmalonic acidemia (PAMMA), and other carnitine related disorders. A total of 69 cases were classified as other acylcarnitine disorders (235 per 100,000 screened newborns), making this the largest subgroup. Specific conditions detected included PAMMA (*n* = 37; 126 per 100,000 screened newborns), IVA (*n* = 15; 51 per 100,000 screened newborns), CPT2/CACT deficiency (*n* = 17; 58 per 100,000 screened newborns), carnitine uptake deficiency (*n* = 4; 13 per 100,000 screened newborns), SCAD (*n* = 6; 20 per 100,000 screened newborns), GA1 (*n* = 2; 6 per 100,000 screened newborns), malonic aciduria (C3dc; *n* = 1; 3 per 100,000 screened newborns), and CPT-1 deficiency (*n* = 1; 3 per 100,000 screened newborns) (Table 4). These disorders, although less common individually, collectively represent a significant portion of the screened metabolic conditions.

**Figure 1:**
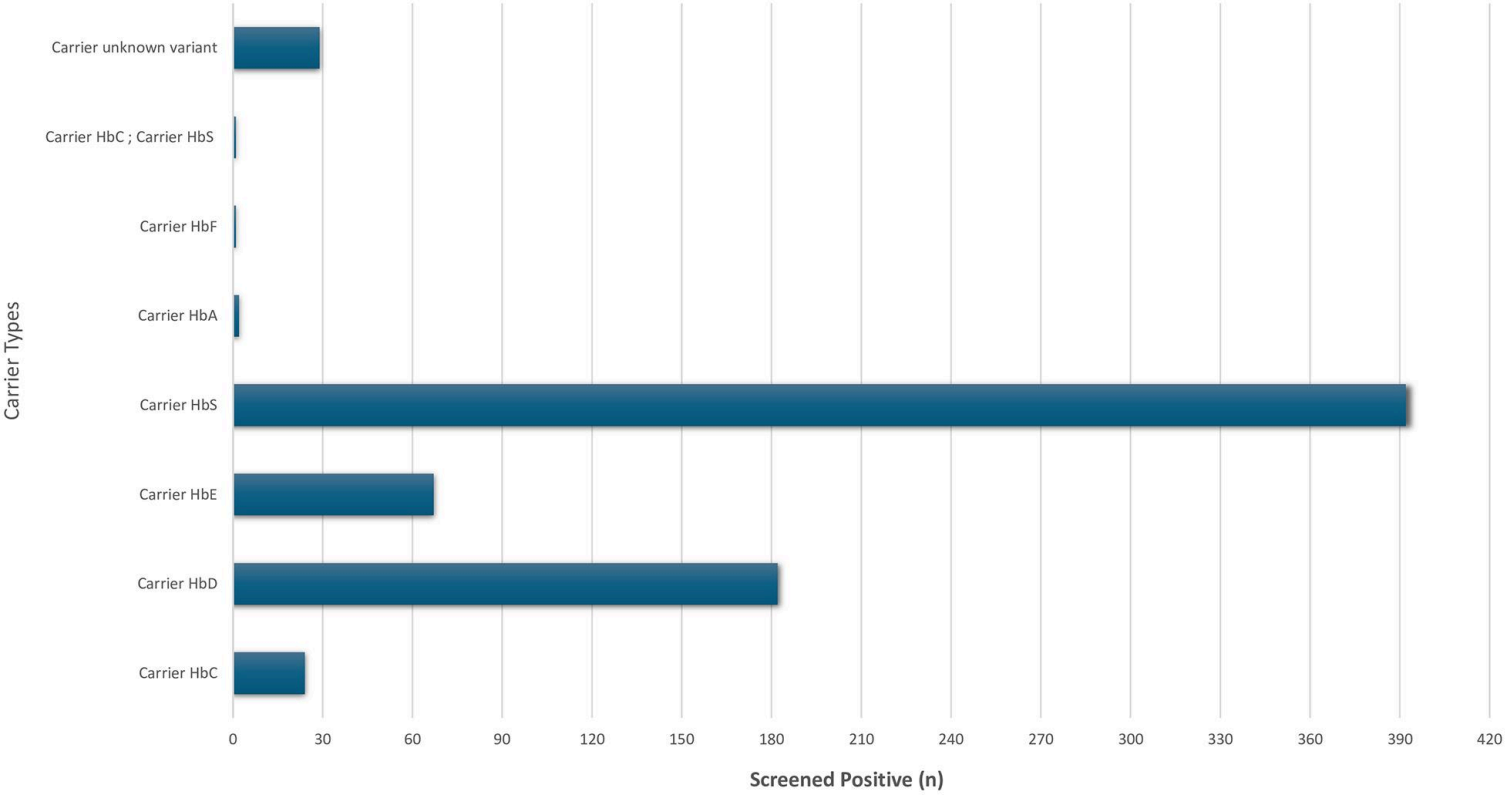
Screened positive carriers of hemoglobinopathies detected during newborn screening in the UAE between 2021 and 2023.

A total of 64 hemoglobinopathy cases were identified during the screening period. Alpha thalassemia was the most prevalent (*n* = 22; 75 per 100,000 screened newborns), followed by sickle cell anemia (*n* = 16; 54 per 100,000 screened newborns) and beta thalassemia (*n* = 8; 27 per 100,000 screened newborns). Less frequent variants included HbC disease (*n* = 2; 6 per 100,000 screened newborns), HbD/beta thalassemia (*n* = 5; 17 per 100,000 screened newborns), HbH disease (*n* = 2; 6 per 100,000 screened newborns), and other hemoglobin disorders (*n* = 9; 30 per 100,000 screened newborns) as shown in Table 4. The category “other hemoglobin disorders” represents unclassified variants detected during screening. In addition to confirmed cases, the program actively screened for hemoglobinopathy carrier states, as illustrated in Figure 1. The highest carrier prevalence was observed for HbS (*n* = 392; 1,338 per 100,000 screened newborns), followed by HbD (*n* = 182; 621 per 100,000 screened newborns), HbE (*n* = 67; 228 per 100,000 screened newborns), and HbC (*n* = 24; 81 per 100,000 screened newborns). Rare carrier states included HbA (*n* = 2; 6 per 100,000 screened newborns), HbF (*n* = 1; 3 per 100,000 screened newborns), and a combined HbC/HbS carrier (*n* = 1; 3 per 100,000 screened newborns) as stated in Table 5. Additionally, 29 carriers with unknown variants (99 per 100,000 screened newborns) were reported. These findings highlight both the presence of clinically significant hemoglobinopathies and a substantial carrier burden in the UAE, underscoring the importance of integrating carrier detection into national newborn screening programs for early genetic counseling and prevention.

**Table 5:** Screened positive carriers and prevalence per 100,000 of hemoglobinopathies detected during newborn screening in the UAE between 2021 and 2023.

Hemoglobinopathy carrier status	Screened positive	Prevalence per 100,000
Carrier HbC	24	81
Carrier HbD	182	621
Carrier HbE	67	228
Carrier HbS	392	1,338
Carrier HbA	2	6
Carrier HbF	1	3
Carrier HbC; Carrier HbS	1	3
Carrier unknown variant	29	99

## Discussion

Newborn screening is a crucial public health strategy that allows pre-symptomatic diagnosis, detection, and the management of congenital conditions in newborns. Newborn screening is vital for maintaining neonates’ health and wellbeing while improving long-term prognosis, which if neglected could lead to severe complications. Each country decides what tests to include in their newborn screening program, the percentage of newborns screened for these disorders differs between countries. In the United States, 100% of the newborns are screened, whereas, in Europe 78%, followed by 28% in the (Middle East and North Africa) MENA region, 13% in Asia and 0% in central African countries. In Africa, about 30,000–40,000 (0.13%) newborns have been screened so far through a pilot study by The Consortium on Newborn Screening in Africa (CONSA) that operates in seven countries Ghana, Kenya, Libya, Nigeria, Tanzania, Uganda, and Zambia. However, this represents only a tiny fraction of ∼30 million babies born annually on the continent (6). The variation in the percentage of newborns screened is partially dependent on the country's financial situation, although it is not the primary cause for the heterogeneity (19). However, UAE being a developed country in the MENA region showed a drastic increase in the percentage of newborns screened from 50% in 1998 to 95% in 2010. This increase in the newborns tested may be due to increased awareness along with education on prenatal health in the country (9). The cut-off values used by the National Reference Laboratory NRL) in the UAE when compared to those used in Prince Sultan Military Medical City (PSMMC) in Saudi Arabia reveals both similarities and differences. PSMMC applies broader cut-offs, while NRL uses more specific, age-stratified thresholds. The UAE panel includes conditions like G6PD deficiency and cystic fibrosis, whereas the Saudi panel covers a wider range of metabolic disorders. For congenital hypothyroidism, the UAE reported an incidence of 1:862 compared to 1:3,775 in Saudi Arabia, potentially reflecting differences in TSH cut-offs. The TSH cut-off threshold of ≥10 mU/L used in our screening protocol (Table 2), which is more sensitive than the commonly used ≥15–20 *μ*U/mL cut-offs in many international programs. UAE uses age-specific values, while PSMMC applies a fixed ≥21 μU/mL threshold. Both use a similar 17-OHP cut-off for CAH (≥60 nmol/L), yet the UAE shows higher prevalence. Galactosemia prevalence is also higher in the UAE as (1:2,128) versus Saudi Arabia (1:56,632), possibly due to differing GALT enzyme thresholds. Biotinidase deficiency is rare in both countries but more frequently reported in the UAE (68 per 100,000 screened newborns) with a stricter cut-off (<30 vs. < 39.4 U/dL). Both programs screen for acylcarnitine and amino acid disorders; however, Saudi Arabia reports propionic aciduria most frequently (1:14,158), while the UAE shows a broader distribution including PAMMA (1:793) and IVA (1:962). The study in Saudia screened about 56,632 newborns born between 2012–2017 when compared to this study in UAE that screened 29,290 newborns who were born between 2021 and 2023 (22). These variations likely reflect both true population differences—such as consanguinity and genetic factors—and differences in cut-offs, screening algorithms, and follow-up protocols (9).

The disorders that the newborns were tested for included Acylcarnitine, aminoacidopathies, biotinidase, CAH, CH, CF, GALT-1, G6PD, and Hb disorders. The results indicated that 92.5% (*n* = 27,099) tests were negative, 7.4% (*n* = 2,191) positive, and about 2.3% (*n* = 698) of the newborns were not affected by the disease but were carriers of the disease-causing allele of hemoglobinopathies. G6PD disorder was of the highest prevalence, followed by Cystic fibrosis, in contrast, the lowest cases were detected in newborns screened for Proximal urea cycle disorder, Carnitine palmitoyl transferase I deficiency (CPT-1), and Malonic Aciduria (C3dc) in the country. Majority of the positive cases for hemoglobinopathies were that of major alpha thalassemia followed by Sickle cell Anemia. The G6PD screening positivity rate (5.3%) likely exceeds confirmed disease prevalence due to detection of heterozygous females with preserved enzyme activity alongside hemizygous males. Genotyping studies are needed to distinguish clinically significant cases from benign carriers.

The overall prevalence rate calculated in our study was higher than that seen in multiple studies, and this could be due to the difference in the total number of newborns screened in each disorder and due to the difference in the total birth per year in UAE when compared to other countries. Another reason for the variability in the prevalence could be due to the high rates of consanguinity in the region. Sampling bias together with random variations may have an impact on the estimated prevalence. Our study indicated about 1:602 (566 per 100,000 screened newborns) cases for CF, whereas a previous study indicated 1:2,618 cases of white population being affected (14). The observed cystic fibrosis screening positivity rate is substantially higher than global estimates of confirmed disease prevalence. This discrepancy is likely due to the nature of the newborn screening algorithm used in this study, which is primarily based on immunoreactive trypsinogen (IRT) screening and identifies screen-positive infants rather than confirmed cystic fibrosis cases. These results therefore reflect screening positivity rates rather than definitive clinical diagnoses, which require confirmatory testing such as sweat chloride analysis and CFTR mutation studies. Variations in screening cut-offs, follow-up confirmation rates, and inclusion of false positives or carriers may also contribute to the higher observed rate. CH cases were reported to be 1:862 (116 per 100,000 screened newborns) cases, whereas a study done in US indicated 1:1,667 newborns (9). Another study was done to find the prevalence of CH in newborns of Saudi Arabia, the study indicated 1:4,208 newborns affected (20). GALT-1 prevalence was seen to be 1:2,128 (47 per 100,000) births which was seen to be higher than those reported in Northern American and the Asian population. The least prevent hemoglobinopathy was seen to be Hb-H and Hb-C with only 2 positive cases each. A study conducted in Saudi Arabia reported 1:4,243 newborns affected by Cystic fibrosis which was lower than what was seen in our study (1:602) newborns affected. The Saudi- Arabia study was comparatively old and only 3 years data was included in our study which could have accounted for the difference in the prevalence (20).

Another study done in UAE for newborn screening cohort reveals notable shifts in disease prevalence when compared to this study. In the 2011–2014 cohort, biotinidase deficiency was the most common inborn error of metabolism (IEM), making up 25% of citizen cases (14/55), whereas its prevalence dropped to 68 per 100,000 screened newborns in the NRL data. Phenylketonuria (PKU), the second most common earlier (20%), showed a significant decline, possibly because the cases in this study are not confirmed by diagnostic technique. Similarly, 3-methylcrotonylglycinuria (16%, 9/55 in the earlier cohort) was less frequent recently, likely reflecting improved screening specificity. Disorders such as MCAD deficiency (7%, 4/55) and organic acidemias (e.g., glutaric aciduria types 1 and 2, 2/55 each) were more prominent in 2011–2014 but lower here, possibly attributable to better early clinical recognition, refined follow-up protocols, and limitations in earlier ascertainment (17).

Newborn screening for hemoglobinopathy disorders enables families to acquire better knowledge of these abnormal hemoglobin traits, helps in determining the risk of recurrence in offspring of affected individuals and carriers (23). Out of the total positive screened cases of hemoglobinopathies, HbS carriers accounted about 56% of all the carriers detected, indicating a high rate of sickle haemoglobin carriers in the country. Test results revealed carriers of HbC, HbD, HbE, HbS, and unknown variants. Although carriers may not show signs of the illness, they can pass the gene on to their children. Carriers for these conditions are often recommended to visit genetic counselors who educate and tell them about the inheritance pattern like autosomal recessive for hemoglobinopathies and risk of passing the disease to their offspring and give them all the possible options on how to reduce the risk (21). This is the first study to be done in the UAE that reports on prevalence of 9 classes of disorders detected by newborn screening. In addition, this study also focusses on the different hemoglobinopathies, and the carriers of different blood disorders detected at birth. Our study contributes significantly to understanding newborn screening procedures in the UAE by evaluating carriers of hemoglobinopathies and emphasizing the use of the heel prick test.

While hereditary factors impact the prevalence of these conditions at birth, the long-term diagnosis and quality of life experienced by affected children are dependent on the severity of the disorder and the availability of appropriate continuing care and treatment. Newborn screening allows early detection, which helps in identifying individuals affected, as well as detecting the carriers of these diseases. It allows to take advantage of early treatment choices that relieve symptoms. Individuals who are diagnosed early have a wider variety of treatment options which can lead to better prognosis in the future. The prevalence data obtained from our study provides a solid basis for future research in this field, particularly in the genetic studies, clinical trials, and epidemiological investigations. It promotes innovation and the creation of novel diagnostic tools, medicines, and preventative means. Furthermore, our findings highlight the need for further research to explore the disease's aetiology and further investigating whether the high number of cases observed in our study is attributable to consanguinity in the region. This study opens new opportunities and paths for expanded research in understanding the disease and its contributing factors.

The major challenging limitation during this study the sample size was small, and the data was retrieved from a single center that receives sample from 20 hospitals and laboratories in UAE, so it cannot be used as to generalize the country's population. Although, some of the newborns were tested for multiple disorders but some of the data was not included due to inconclusive and Invalid results. Data was collected from multiple healthcare facilities across the United Arab Emirates, including Abu Dhabi, Dubai, Sharjah, and Fujairah with single-center laboratory analysis (NRL as central processing lab). The data we received included only the main emirates while, smaller emirates like Ajman, Ras-al-Khaimah and Um-al -Quwain were not included in the study, these populations were underrepresented or could have been tested in those 20 centres. This may enhance standardization but may still limit full generalizability and site-specific interpretation. As this was a retrospective study based on existing newborn screening records, the analysis was dependent on the completeness and accuracy of recorded data. This may introduce information bias due to missing or incomplete documentation, particularly for certain variables such as ethnicity. Ethnicity was often not recorded, which is a key factor in haemoglobinopathies. This is a lab-based data set which is linked to the hospital systems only for a specific test. The ethnicity data lies with the hospital electronic health record system and cannot be accessed through the associated lab. As a result, our findings should be taken with caution as they do not generalize other facilities in the country, and further research needs to be done to find if our findings are due to chance or have significance to them. Another similar study with confirmatory test results for same individuals can be done in future. In addition, G6PD deficiency prevalence (5.3%; 5,278 per 100,000) represents screening positivity rather than confirmed hemizygous males/compound heterozygous females, as genotype-specific confirmation (PCR for specific variants) was unavailable. This likely overestimate true disease prevalence by including heterozygous females with normal enzyme activity, limiting comparability with genotype-confirmed studies. The study is limited by its descriptive design and relatively small sample size within several subgroups, which precluded the use of robust inferential statistical analyses, including confidence interval estimation and formal tests for temporal trends. Future studies with larger sample sizes and longer follow-up periods are recommended to allow for more comprehensive statistical evaluation.

Further research can be done improve the representativeness and accuracy of study findings by improving sample collection procedures. Sample collection procedures could be improved by increasing the sample size to include a larger and more varied population, for e.g., using data from all the newborns from all the emirates in the country. Furthermore, patient details could be taken during data collection to ensure that the patients belonged to the emirate from which they got tested from. Long-term follow-up of the screened newborns is a recommendation for the future as it helps in analysing the outcomes and consequences of the confirmed cases. Follow-up data is useful for determining disease progression, effectiveness of therapy, and long-term health implications. Governmental authorities can take an initiative in ensuring the follow-up of these carriers and to make genetic screening mandatory for these patients. This in-turn educates the carriers and provides them with a variety of reproductive options that can help in reducing the genetic burden in these families. There is a need for structured newborn screening registries with proper follow-up systems to ensure continuity of care for children with genetic conditions. Newborn screening alone is insufficient without long-term management. A multidisciplinary approach involving geneticists, paediatricians, haematologists, endocrinologists, and genetic counselors is essential to provide comprehensive care and improve patient outcomes. Our study is helpful in identifying the common congenital deficiencies and aims to provide awareness on the importance of screening programs that could help in early diagnosis, intervention, healthcare planning, and public health monitoring. These findings provide crucial data that can benefit individual newborns, families, healthcare system, and society as a whole.

## Conclusion

This study provides a comprehensive overview of newborn screening outcomes in the UAE, based on 29,290 screened newborns between 2021 and 2023. A total of 2,191 newborns were identified as screen-positive, with glucose-6-phosphate dehydrogenase (G6PD) deficiency showing the highest prevalence (5,278 per 100,000 screened newborns), followed by cystic fibrosis, acylcarnitine disorders, and aminoacidopathies. Hemoglobinopathies were identified in 218 per 100,000 screened newborns, alongside a substantial carrier burden (*n* = 698), highlighting the importance of incorporating carrier detection into screening programs. These findings indicate a considerable burden of genetic and metabolic disorders in the screened population and reinforce the value of newborn screening as an essential public health strategy for early detection and intervention. However, the results primarily reflect screening positivity rather than confirmed diagnoses, emphasizing the need for robust confirmatory testing and structured follow-up systems. Strengthening national newborn screening registries, ensuring long-term follow-up, and adopting a multidisciplinary care approach involving geneticists, clinicians, and genetic counselors are critical to improving patient outcomes. Overall, this study provides important population-level data to inform healthcare planning and future research in genetic disease prevention and management in the UAE.

## Data Availability

The original contributions presented in the study are included in the article/Supplementary Material, further inquiries can be directed to the corresponding authors.

## References

[1] Dubay KS Zach TL. Newborn screening. In: Shams P, editor. StatPearls. Treasure Island (FL): StatPearls Publishing (2022).

[2] Yau M Gujral J New MI. Congenital adrenal hyperplasia: diagnosis and emergency treatment. In: Feingold KR Anawalt B Boyce A Chrousos G Dungan K Grossman A Wilson DP, editors. Endotext. South Dartmouth (MA): MDText.com, Inc (2000). 25905311

[3] Pitt JJ. Newborn screening. The Clinical Biochemist. Reviews. (2010) 31(2):57–68. 20498829 PMC2874432

[4] Mujamammi AH. Insights into national laboratory newborn screening and future prospects. Medicina (Kaunas, Lithuania). (2022) 58(2). 10.3390/medicina58020272 PMC8879506 35208595

[5] Therrell BL Padilla CD Loeber JG Kneisser I Saadallah A Borrajo GJC. Current status of newborn screening worldwide: 2015. Semin Perinatol. (2015) 39(3):171–87. 10.1053/j.semperi.2015.03.002 25979780

[6] Twum S Fosu K Felder RA Sarpong KAN. Bridging the gaps in newborn screening programmes: challenges and opportunities to detect haemoglobinopathies in Africa. Afr J Lab Med. (2023) 12(1):2225. 10.4102/ajlm.v12i1.2225 38116518 PMC10729498

[7] A Hosani H Salah M Osman HM Farag HM El-Assiouty L Saade D. Expanding the comprehensive national neonatal screening programme in the United Arab Emirates from 1995 to 2011. Eastern Mediterranean Health Journal. (2014) 20(1):17–23. 10.26719/2014.20.1.17 24932929

[8] Cellucci MF. Newborn Screening Tests. Accessed March 20, 2023 (2022). Available online at: https://kidshealth.org/en/parents/newborn-screening-tests.html

[9] Ata H. 152,074 newborns screened for genetic, congenital disorders | Uae – Gulf News. (2017). Accessed May 9, 2023, Available online at: https://gulfnews.com/uae/152074-newborns-screened-for-genetic-congenital-disorders-1.68347231

[10] Babar V. Genetic Testing Market. California: Meticulous Market Research (2023).

[11] Al-Gazali LI Bener A Abdulrazzaq YM Micallef R Al-Khayat AI Gaber T. Consanguineous marriages in the United Arab Emirates. J Biosoc Sci. (1997) 29(4):491–7. 10.1017/s0021932097004914 9881148

[12] McCandless SE Wright EJ. Mandatory newborn screening in the United States: history, current status, and existential challenges. Birth Defects Research. (2020) 112(4):350–66. 10.1002/bdr2.1653 32115905

[13] Health of vulnerable groups. The Official Portal of the UAE Government. (n.d.). Accessed June 5, 2023, Available online at: https://u.ae/en/information-and-services/health-and-fitness/health-of-vulnerable-groups#:∼:text=Department%20of%20Health%20in%20Abu%20Dhabi%20introduced%20the,screening%20Congenital%20heart%20disease%20screening%20Heel%20prick%2Fbloodspot%20test

[14] Feuchtbaum L Carter J Dowray S Currier RJ Lorey F. Birth prevalence of disorders detectable through newborn screening by race/ethnicity. Genet Med. (2012) 14(11):937–45. 10.1038/gim.2012.76 22766612

[15] Huang X Zhang Y Hong F Zheng J Yang J Tong F. [Screening for amino acid metabolic disorders of newborns in zhejiang province:prevalence, outcome and follow-up]. Zhejiang Da Xue Xue Bao Yi Xue Ban. (2017) 46(3):233–9. 10.3785/j.issn.1008-9292.2017.06.02 29039163 PMC10396939

[16] Golbahar J Al-Jishi EA Altayab DD Carreon E Bakhiet M Alkhayyat H. Selective newborn screening of inborn errors of amino acids, organic acids and fatty acids metabolism in the kingdom of Bahrain. Mol Genet Metab. (2013) 110(1-2):98–101. 10.1016/j.ymgme.2013.07.006 23916421

[17] Al-Jasmi FA Al-Shamsi A Hertecant JL Al-Hamad SM Souid A-K. Inborn errors of metabolism in the United Arab Emirates: disorders detected by newborn screening (2011-2014). JIMD Rep. (2016) 28:127–35. 10.1007/8904_2015_512 26589311 PMC5059198

[18] A Hosani H Salah M Abu-Zeid H Farag HM Saade D. The national congenital anomalies register in the United Arab Emirates. Eastern Mediterranean Health Journal. (2005) 11(4):690–9. 16700385

[19] Aktuğlu Zeybek AÇ. Newborn screening: from the past to the future. Turkish Archives of Pediatrics. (2022) 57(5):473–5. 10.5152/TurkArchPediatr.2022.16082022 36062438 PMC9524414

[20] Bashir A. Towards a uniform newborn screening panel in the kingdom of Saudi arabia. American Journal of Psychiatry and Neuroscience. (2018) 6(5). 10.19080/AJPN.2018.06.555753

[21] Kladny B Williams A Gupta A Gettig EA Krishnamurti L. Genetic counseling following the detection of hemoglobinopathy trait on the newborn screen is well received, improves knowledge, and relieves anxiety. Genet Med. (2011) 13(7):658–61. 10.1097/GIM.0b013e31821435f7 21546841

[22] Mohamed S Elsheikh W Al-Aqeel AI Alhashem AM Alodaib A Alahaideb L. Incidence of newborn screening disorders among 56632 infants in central Saudi Arabia. A 6-year study. Saudi Med J. (2020) 41(7):703–8. 10.15537/smj.2020.7.25147 32601637 PMC7502916

[23] Williams TN Thein SL. Sickle cell anemia and its phenotypes. Annu Rev Genomics Hum Genet. (2018) 19:113–47. 10.1146/annurev-genom-083117-021320 29641911 PMC7613509

